# Coronas of micro/nano plastics: a key determinant in their risk assessments

**DOI:** 10.1186/s12989-022-00492-9

**Published:** 2022-08-06

**Authors:** Jiayu Cao, Qing Yang, Jie Jiang, Tatenda Dalu, Aliaksei Kadushkin, Joginder Singh, Rawil Fakhrullin, Fangjun Wang, Xiaoming Cai, Ruibin Li

**Affiliations:** 1 School of Public Health, Soochow University, Suzhou, 215123 Jiangsu China; 2 State Key Laboratory of Radiation Medicine and Protection, Collaborative Innovation Center of Radiological Medicine of Jiangsu Higher Education Institutions, School for Radiological and Interdisciplinary Sciences (RAD–X), Soochow University, Suzhou, 215123 Jiangsu China; 3 School of Biology and Environmental Sciences, University of Mpumalanga, Nelspruit, 1200 South Africa; 4 Department of Biological Chemistry, Belarusian State Medical University, 220116 Minsk, Belarus; 5 Department of Microbiology, Lovely Professional University, Phagwara, Punjab 144411 India; 6 Kazan Federal University, Institute of Fundamental Medicine & Biology, Kreml Uramı 18, Kazan, Republic of Tatarstan Russian Federation 420008; 7 Dalian Institute of Chemical Physics, Chinese Academy of Sciences (CAS), Dalian, 116023 Liaoning China

**Keywords:** Micro/nanoplastics, Nanotoxicity, Corona, Structure–activity relationship, Biotransformation

## Abstract

As an emerging pollutant in the life cycle of plastic products, micro/nanoplastics (M/NPs) are increasingly being released into the natural environment. Substantial concerns have been raised regarding the environmental and health impacts of M/NPs. Although diverse M/NPs have been detected in natural environment, most of them display two similar features, i.e.,high surface area and strong binding affinity, which enable extensive interactions between M/NPs and surrounding substances. This results in the formation of coronas, including eco-coronas and bio-coronas, on the plastic surface in different media. In real exposure scenarios, corona formation on M/NPs is inevitable and often displays variable and complex structures. The surface coronas have been found to impact the transportation, uptake, distribution, biotransformation and toxicity of particulates. Different from conventional toxins, packages on M/NPs rather than bare particles are more dangerous. We, therefore, recommend seriously consideration of the role of surface coronas in safety assessments. This review summarizes recent progress on the eco–coronas and bio-coronas of M/NPs, and further discusses the analytical methods to interpret corona structures, highlights the impacts of the corona on toxicity and provides future perspectives.

## Introduction

Plastic is one of the most important engineered materials discovered in all aspects of life [1]. It is an ideal substance that is used to manufacture household, commercial and industrial products [2, 3]. Plastic products are widely present in our daily life, and a major portion of them are disposable packaging or single–use items that are discarded within a short period of time [4]. The global production of plastic is growing rapidly and reached 368 million metric tons in 2019. It has been estimated that 33 billion tons of plastics will be produced by 2050 [5], whereas global plastic waste is expected to grow to 155–265 million metric tons by 2060 [6]. Currently, the end–of–life fate of 74–94% of discarded plastics is landfills, incinerators, and/or the natural environment, leading to the accumulation of plastic waste within the environment [7]. Once released into the environment, plastics persist for many years and gradually breakdown into small debris, i.e.,microplastic (< 5 mm) and nanoplastic (< 100 nm) debris [8, 9], via natural degradation (e.g., hydrolysis, biodegradation, photodegradation, wind, water erosion). In addition, some micro/nanoplastics (M/NPs) are deliberately engineered for commercial and domestic use in cosmetics [10], personal care products [3] and pigments [11] and may be directly released into the natural environment. Due to their small sizes, M/NPs may spread into diverse media, including air, lakes, rivers, oceans and soil, interacting with organisms in the ecosystem and further causing adverse outcomes [12].

Although over 30,000 types of plastic polymers have been manufactured and applied in real scenarios, the resulting M/NPs have two similar features, *i.e.,*high surface area and strong binding affinity, which enable extensive interactions between M/NPs and other substances such as metal cations, inorganic anions, organic chemicals and biomolecules [13]. These substances may be adsorbed on the surfaces by hydrophobic interactions with the C–C/C = C skeletons or electrostatic forces with the functionalities (e.g., –OH, –COOH, –HSO_3_, –NH_3_) of M/NPs. The adsorbed substances on fine particulates are defined as “corona”, consisting of a “hard” and a “soft” layer [14]. The “hard” corona often consists of a monolayer of bound molecules that tightly associate on the particle surface and form in the initial transient period (seconds to minutes) of interaction with surrounding media [15]. This layer is often long lived and relatively stable because the constituents in this layer are difficult to replace with new molecules when the particles transfer to a new environment. The “hard” corona is then covered by loosely bound molecules, which are termed the “soft” corona. In contrast to the “hard” corona, the constituents of the soft corona are unstable and may rapidly exchange with environs [16]. The coronas on M/NPs could be classified into two categories, eco-corona and bio-corona, in terms of the settling circumstances (*i.e.,*abiotic environmental media and living organisms) of M/NPs [17]. The difference in corona structures of M/NPs in culture media and physiological fluids [18] (*e.g., *blood, gastric, intestinal and bronchoalveolar lavage fluids) may result in distinct hazard effects, but this has rarely been explored. While metal ions, inorganic anions and organic chemicals mainly account for the compositions of eco–corona [19], bio-coronas are composed of proteins, lipids, nucleic acids, amino acids, ribonucleic acid (RNA) and deoxyribonucleic acid (DNA) [20].

In risk assessments of fine particulates, coronas play a critical role in adverse outcome pathways, such as hemolysis [21], thrombocyte activation [22], biodistribution [23], immune response [24], reactive oxygen species (ROS) production [25], cellular uptake [26], and cell death [22]*.* For instance, the formation of a serum–derived corona on some nanoparticles (*i.e.,*silica [27], iron oxide [28], carbon [29], polystyrene [30]) could reduce their cellular internalizations. Deng et al*.* [31], reported that negatively charged poly (acrylic acid)–coated gold nanoparticles could enrich unfolded plasma fibrinogen, which enables strong binding to the membrane Mac–1 receptor, resulting in an inflammatory response by NF–κB activation. In terms of the real exposure scenarios, there are limited accesses for bare M/NPs to interact with biological systems. However, previous toxicity studies on M/NPs ignored or failed to consider the impacts of surface coronas.

Recently, substantial research interest has been directed to the environmental and health impacts of M/NPs [32, 33]. The number of studies has increased at 30–50% annually in the past five years [34, 35]. It could be expected that more research will be directed towards the subject area. Before considerable efforts are put forward in behavior investigation, safety assessment and treatment of M/NPs, it is important to highlight the key factors that determine their fates in the natural environment. Micro/nanoplastics themselves are typically considered inert and stable materials because their basal skeletons are hard to break [36], suggesting that these materials have limited access to directly interact with key mediators in living organisms. In contrast, corona formation on M/NPs is inevitable and often displays variable and complex structures, which may alter the physicochemical properties of M/NPs as well as their environmental/biological behaviors [37]. From this perspective, we reviewed recent progress on eco- and bio-coronas of M/NPs, discussed corona impacts on toxicity, summarized the analytical methods to interpret corona structures, and provided future perspectives on M/NPs coronas.

## Matrices and physicochemical properties of environmental micro/nanoplastics

**Fig. 1:**
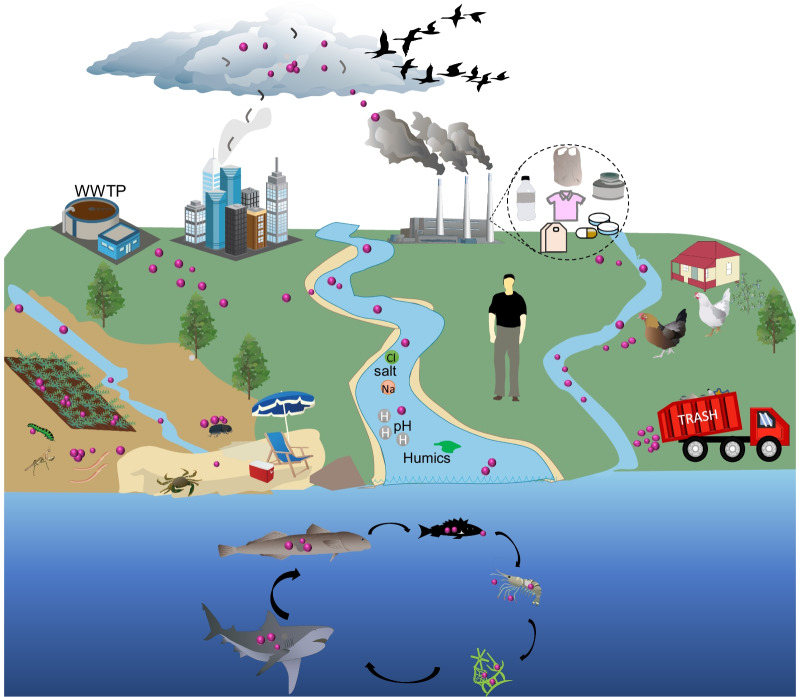
The production, migration and distribution of M/NPs in the environment. Plastics are released to the environment during manufacture, use and disposal. The environmental plastics can be gradually broken down into M/NPs. M/NPs can easily diffuse elsewhere through water and/or air circulation. M/NPs have been found to be widespread in air, lakes, rivers, oceans and soil, where they can be taken up by various organisms.

Global production and mismanaged plastic waste lead to a magnitude of M/NP contamination in the environment [38]. Owing to their small size, M/NPs can easily diffuse elsewhere through water and/or air circulation [39], as shown in Fig. 1. They have been found to be widespread in water [40], soil [41] and air [42], where they can be taken up by plants and animal organisms, with serious detrimental effects [43]. Since the first plastics were made from fossil fuels, a large number of plastic types have been developed for various applications [44]. According to the data collected by PlasticsEurope, more than 30,000 different plastic polymers are registered in the European Union [45]. As a result, the chemical composition, size, shape and surface properties of observed plastics in the environment usually vary significantly.

### Matrices contaminated by micro/nanoplastics

#### Waters

Micro/nanoplastics are ubiquitous in the water environment, especially in oceans. From 4.8 to 12.7 million metric tons of mismanaged plastic waste is released to the ocean every year [46]. According to Wayman et al. [47],the number of plastics released into the ocean will grow by 2.6–fold between year 2016 to 2040. In addition, freshwater has been considered as one of the origins and transport pathways of ocean plastic waste [48]. Since the first reported microplastics (MPs) in a UK freshwater system by Horton et al*.* [49], MPs have been detected in various freshwaters, such as Danube River [50], Ottawa River [51], Yangtze River [52], Erie Lake [53] and Taihu Lake [54]. In addition, plastic particles have been found in drinking water from Changsha, China at range abundances of 2173–3998 (mean = 2753.0), 338–400 (mean = 351.9), and 267–404 (mean = 343.5) particles per liter in freshwater, treated water, and tap water, respectively [55]. The M/NPs released in waters may be taken up by aquatic organisms, such as Echinodermata [56], mollusks [57], crustaceans [58] and fish [59].

#### Soil

Although less attention has been received, soil is regarded as a larger reservoir for M/NPs than the ocean [60]. Microplastic contamination on land was estimated to be 4–23–fold larger than that of oceans [61]. Soils may be contaminated by M/NPs from wastewater irrigation [62], plastic mulching [63], sludge utilization [64], atmospheric deposition [65], road runoff [66]. Indeed, M/NPs have been detected in floodplains, coastal beaches, and agricultural soils. For instance, MPs are found in 90% of Swiss floodplain soils, with a range of 5 mg/kg to 55.5 mg/kg [67]. Zhou et al*.* [68] reported 1.3–14,712.5 particles/kg of MPs in coastal beach soils from Shandong, China. In another report, 78.00 ± 12.91 and 62.50 ± 12.97 particles/kg of MPs were detected in shallow and deep farmland soils from 20 vegetable fields around the suburbs of Shanghai, China [69]. In addition, accidental loss or improper handling of plastic debris in landfills and urban and industrial centers may result in the input of M/NPs in land soil [70]. Accumulated M/NPs have also been found in plants [71] and worms [72].

#### Air

Atmospheric M/NPs have aroused substantial concerns in recent years because they can be quickly transported over long distances by wind. Airborne plastics mainly originate from synthetic clothing, synthetic fibers in building materials, plastic waste incineration and road dust [73]. Researchers have found atmospheric M/NPs both indoors and outdoors [74]. Most of the detected atmospheric M/NPs are fibers, and the concentrations of fibers suspended in indoor environments are significantly higher than those outdoor areas [75]. Although the reported sizes of atmospheric plastics are 2–9555 µm, nanoplastics (NPs) may account for a large portion because they are more stable in airborne environs and can be suspended in air for long periods. This, therefore, highlights that reliable and sensitive detection techniques for airborne NPs are urgently needed. These fine particulates have high respiratory exposure risks and may reach the lower airways and pass through the air–blood barrier of mammals and birds [76], resulting in severe pulmonary diseases such as lung fibrosis and pleural granuloma formation [77].

#### Diet

Currently, it is well known that plastic particles can be taken up by aquatic and terrestrial organisms and thus enter the food chain. The impact of plastic debris on marine life has been extensively studied [78]. It has been revealed that over 1 million marine animals die each year due to plastic debris in the ocean [79]. Recently, a meta-analysis estimated the level of microplastic contamination in seafood and the amount of microplastic people may ingest each year, for example, microplastic abundances in mollusks, crustaceans, fish, and echinodermata were 10.5, 8.6, 2.9 and 1 particles/g, respectively [80], with a maximum human uptake of MPs from seafood of ~ 55,000 particles annually. Although most of the ingested M/NPs are excreted by the peristalsis of GI tract [81, 82], some M/NPs may cross the intestinal barrier and spread into other organs (e.g., liver, spleen, blood) [83]. For instance, nylon MPs could be completely excreted from rats by feces in 48 h [83]. Forty-five percent of fluorescent polypropylene NPs were excreted from zebrafish after 24 h depuration [82], which is similar to the behavior of polystyrene NPs in scallops with half-life of elimination in 1.4 days [84]. The excretion rates may closely relate to the composition, size and shape of M/NPs. For example, Hoang et al*.* [81] found that particles with round smooth shape are excreted faster than pointy shaped particles or fibers in *Pimephales promelas*. In addition, microplastic contamination has also been detected in other foods such as beverages, honey, and salt [85]. Moreover, food or drink containers may release substantial amounts of M/NPs during their use [86]. Recently, Su et al*.* [87] detected numerous flake– or oil–film–shaped M/NPs (0.6–332 μm) during the sterilization of feeding bottles. Interestingly, one–year bottle feeding will result in an ingestion of ca. 660,000 MPs by infants.

### Physicochemical properties of micro/nanoplastics

#### Compositions

Despite the large number of plastic polymers manufactured, there are six commonly used namely polyethylene (PE), polypropylene (PP), poly (vinyl chloride) (PVC), polyurethane (PUR), poly (ethylene terephthalate) (PET) and polystyrene (PS) which account for approximately 81.2% of the global plastic demand in 2019 [88]. The building blocks of all six plastic materials are polymers with long chains of covalent–bonded atoms. The backbones of PE, PP, PVC and PS are carbon chains, which are nonbiodegradable [89]. The PE is merely made up of an ethyl chain, while PP, PVC and PS have methyl, chloride and phenyl side groups on the carbon skeleton, respectively. The PE can be further divided into high–density PE (HDPE), low–density PE (LDPE) and linear low–density PE (LLDPE) according to the degree of branching in the polymer [90]. Due to its wide application and poor biodegradability, PE has been reported to be the most abundant plastic accumulated in the environment [91]. The fourth and fifth most–produced polymers PUR and PET contain carbamate (–COONH–) linkage and ester groups, respectively, which are supposedly to be more susceptible to degradation by natural microorganisms. However, PUR and PET degradation in the natural environment is slower than the expected level, and substantial amounts of PUR and PET M/NPs still accumulate in the environment [92]. Besides the raw polymers, plasticizers, such as phthalates, trimellitates, citrates are commonly added into the polymers to improve the performances of plastic products. During the degradation or treatment of plastics, these additives may be released into the environment and co-exist with M/NPs. Since the addition of plasticizers could significantly enhance the physicochemical properties (*e.g., *stability and flexibility) of plastic products [93], these additives may alter the surface chemistry of M/NPs, and consequently impact the formation of surface coronas as well as the interactions with biological systems [94].

#### Shapes

**Fig. 2:**
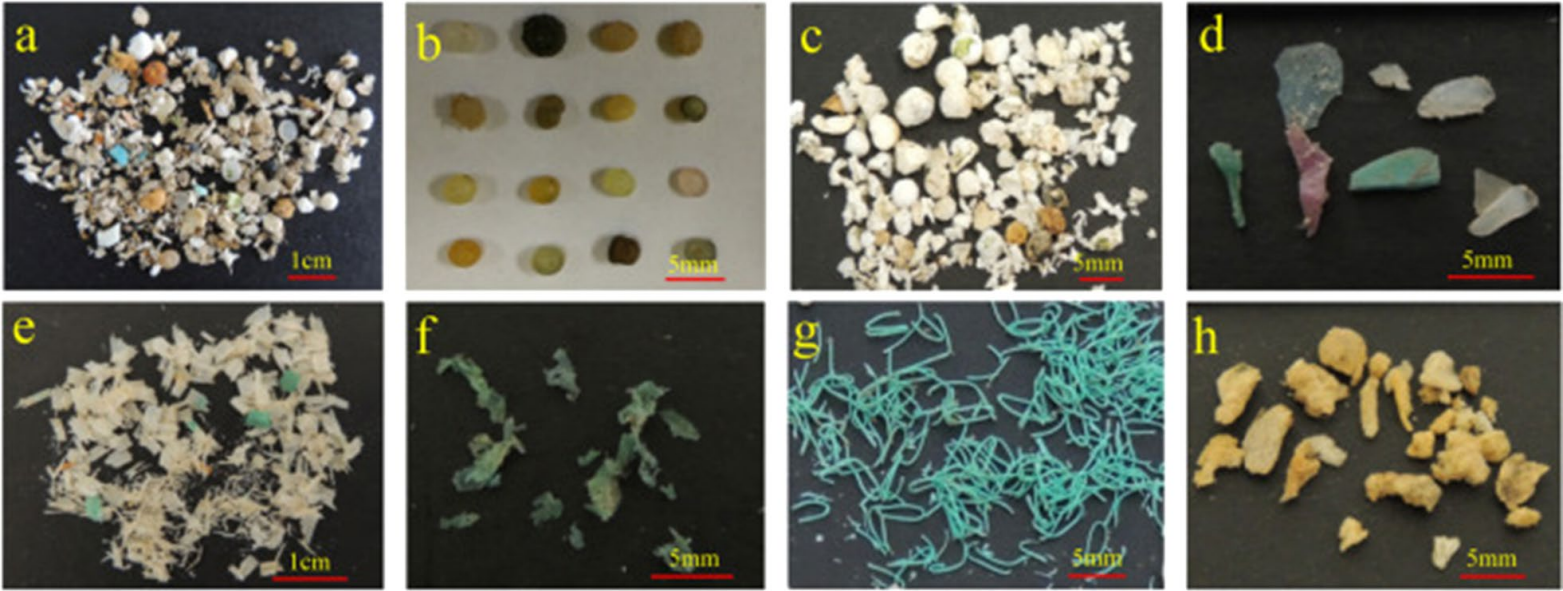
Typical shapes of environmental M/NPs. **a** mixed M/NPs, **b** pellets, **c** foams, **d** fragments, **e** flakes, **f** films, **g** fibers (fishing lines), **h** sponges. Reprinted with permission from [68]

Environmental M/NPs can be classified by their shape into beads, fragment fibers, films, flakes, sponges, foam (Fig. 2) [68]. Generally, microplastic pellets are hard, regular, discoid– or cylindrical–shaped, while fragments are described as hard, irregular and jagged particles. Micro/nanoparticle fibers are described as long, thin, and having a smooth linear morphology. The films are very thin, transparent and soft, the flakes are flat sheets, and the foam and sponges are usually lightweight and porous plastics [68]. However, the definition of the actual shapes of each category of M/NPs is still highly diverse. In addition, these descriptive shape categories are often meaningless for studying particle fate. In view of this situation, Kooi et al*.* [95] recently proposed using length:width:height (L:W:H) ratios as a more generalized approach for different shape categories. According to this approach, the W:H ratio for fragments, foams and films should be ≤ 1, the L: W = H ratios for fibers vary between 0.50 and 0.001, and the L:W:H ratios range from 1 to 0.36 for perfect spheres or ellipsoidal particles.

The shape of environmental plastic debris is related to its origin and history of degradation in the environment. For example, fibrous MPs are reported to be the most encountered shape in atmospheric particulates, as synthetic fibers in clothes and building material are the main source for airborne plastics [96]. On the basis of their category approach, Kooi et al*.* [95] estimated that the most abundant M/NPs in water and sediment are fibers (48.5%), followed by fragments (31%), beads (6.5%), films (5.5%), and foam (3.5%). Several studies on coastal soils also reported that foams or fibers were the predominant microplastic types [97]. However, Zhou et al*.* [68] revealed that the most abundant shape category of microplastics in coastal soils of the Bohai Sea and the Yellow Sea is flakes (69.0%), followed by foams (27.8%), fragments (1.1%), fibers (1.0%), sponges (0.8%), films (0.2%) and pellets (0.1%). Therefore, the shape distributions of M/NPs may vary greatly among different environmental matrices and regions.

#### Sizes

Environment plastics are available in a wide range of sizes and are usually classified by their sizes into macroplastics (> 25 mm), meso–plastics (5–25 mm) and MPs (< 5 mm) [98]. Microplastics can be further divided into large MPs (1–5 mm) and small MPs (< 1 mm) according to the Guidance on Monitoring of Marine Litter in European Seas of the EU Marine Strategy Framework Directive (MSFD). From an analytical point of view, Hidalgo–Ruz et al*.* [99] suggested differentiating MPs of 500 μm–5 mm and < 500 μm since the first fraction is suitable for visual sorting and spectroscopic techniques are required to differentiate the second category. Numerous studies have demonstrated the ubiquitous presence of the two size fractions of microplastics in the environment, including water [100], soil [60] and air [101]. The measurement of MPs with different size ranges has also been summarized by a series of review articles [102].

Recently, NPs have emerged as a hot topic, as the weathering of macroplastics and MPs may lead to a considerable burden of NPs in the environment. Unlike MPs, the size definition of NPs is still under debate [103]. Although nanoplastics are generally defined as particle sizes < 100 nm, which follows the threshold commonly used in engineered nanomaterials [104], Gigault et al*.* propose a definition of nanoplastics with the size range from 1 to 1000 nm because nanoplastics at this range present a colloidal behavior [105]. This debate on the definition of NPs may continue for years, even decades. Regardless of the conflict on size definition, the hazard and exposure risks of NPs have attracted more attention as NPs are more extensively distributed and detrimental than MPs [106].

#### Surface charges

The surface of plastics can be engineered with different moieties to improve their stability [107], adhesion [108] and biocompatibility [109]. Debris of these functionalized plastics may preserve the engineered functionalities and display different surface charges [110]. Moreover, diverse chemical groups may arise on the surfaces of M/NPs during their degradation and migration in the environment. Currently, reactive functional groups, including carboxylic, phenoxy hydroxyls, alcoholic hydroxyls, carbonyl, hydroxides, amine, and sulfonic groups [111], have been widely identified in environmental polymers. These functionalities of M/NPs result in different surface charges, impacting their corona formation, environmental and biological fates and toxicity.

## Corona formation on micro/nanoplastics

**Fig. 3:**
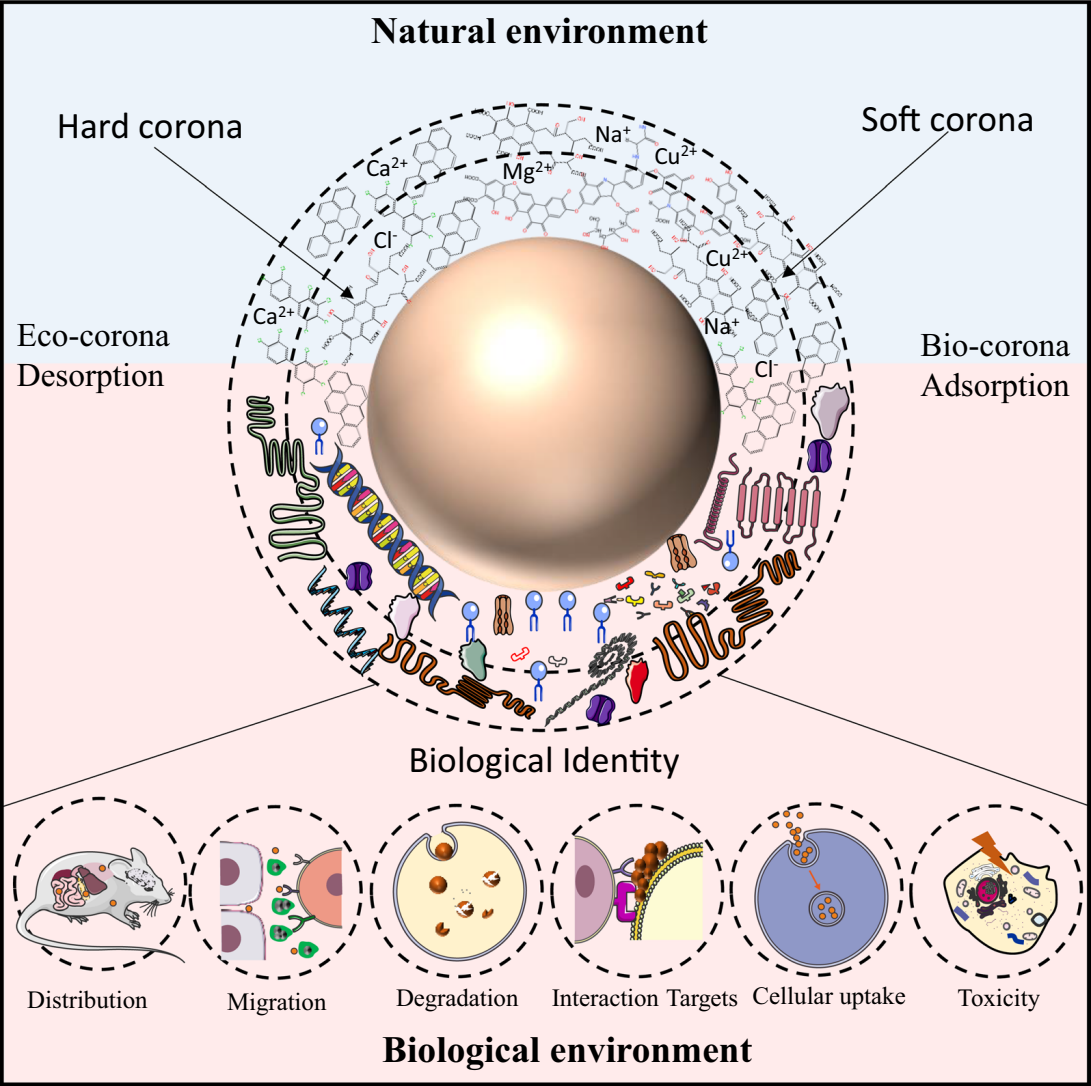
Eco-coronas (top) and bio-coronas (bottom) on M/NP. The natural components in the environment can be adsorbed on the surface of M/NP and form eco-coronas, which consist of metal ions (Cu^2+^, Mg^2+^, Pd^2+^, etc*.*), inorganic salts (Na^+^, Cl^−^, etc*.*), natural organic matters (HAs, FAs, etc*.*), and persistent organic pollutants (PCBs, PAHs, etc*.*). When this M/NP encounters biological systems, it may interact with abundant biomolecules, including proteins, lipids, and nucleic acids to replace some of the constituents in eco-coronas and form bio-coronas on surface. The corona structures offer new environmental or biological identities for M/NP and impact its distribution, migration, degradation, interaction targets, cellular internalization and toxicity in organisms. Adapted and reprinted with permission from [120].

In addition to the primary properties, M/NPs may display secondary properties in media, such as agglomeration and corona formation. Both the primary and secondary physicochemical properties of particulates have been considered to play a decisive role in their biological effects [112]. Alternations of the physicochemical properties in media, *i.e.,*biotransformation often results in molecular initiating events that further elicit adverse outcomes [113, 114]. Given that bare M/NPs are extremely stable and inert, corona formation is the key event that may impact the transportation, cellular internalization, biodistribution and elimination of M/NPs in biological systems [115]. In the migration paths of M/NPs, they mainly encounter two types of media, including environmental media (*e.g., *rivers, lakes, ocean, soil) and biological fluids (*e.g., *saliva, gastric, intestinal and lung lining / alveolar fluids, blood), and form eco–coronas and bio-coronas, respectively (Fig. 3). The corona structures offer new environmental or biological identities for M/NPs, resulting in different distributions [116], migration [117], degradation [118], cellular internalization [115], interaction targets [118]. In biological systems, corona formation on M/NP surfaces may result in three types of adverse effects, *i.e.,*antagonism, synergism and independent action [119]. If the combined effect of corona constituents and M/NPs is greater than their individual toxicity, it is defined as a synergistic effect. Otherwise, the antagonistic effect is regarded. Independent action refers to the effect that M/NPs and corona constituents interact with their own targets by independent signaling pathways without crosstalk.

### Eco–coronas

Inorganic salts and humic acids (HAs) are the major natural components in environmental media [121]. In addition, due to increases in human activities, some engineered chemicals, especially persistent organic pollutants, have been extensively released into the environment [122]. These species may be adsorbed on the surfaces of M/NPs and form an “eco–corona”. Several studies have indicated that the eco–corona is involved in the degradation, migration and toxicity of M/NPs [123]. For instance, studies have shown that environmentally exposed microplastic particles were internalized significantly more often than pristine microplastic particles into macrophages [115]. Meanwhile, the formation of eco–corona on M/NPs may alter the behaviors of environmental pollutants, such as heavy metals and persistent organic pollutants (POPs). A study on the amphipod *Talitrus saltator* demonstrated that ingestion of contaminated MPs (polybrominated diphenyl ether) transferred organic pollutants to its tissues. In contrast, pristine MPs ingested by an amphipod contaminated by organic pollutants removed the pollutants from the tissues [124].

#### Metal cations

Since most M/NPs display a negative surface charge, electrostatic binding is the major mechanism for the interactions between M/NPs and heavy metal cations. Metal ions often bind with polar surface functionalities, such as –COOH, –NH_2_, and phenyl–OH. Holmes et al. [125] studied M/NPs in estuarine conditions and observed greater metal adsorption (at least an order of magnitude greater) on beached pellets *vs.* virgin pellets because the former M/NPs had more surface functional groups. The adsorption of metals to polymers increases with material aging, and its polarity, surface area and porosity increase [126]. In addition, M/NPs may first adsorb a layer consisting of biomolecules or natural organic matter (NOM), resulting in charged surfaces for electrostatic binding with metal ions [127]. Rochman et al*.* [128] found that M/NPs suspended in seawater for a year enriched similar levels of metals irrespective of different compositions, indicating that the accumulation of metals on M/NPs may be mediated by the organic corona layer. A recent study indicated that the corona layer not only intensified the vector role of MPs in the migration of heavy metals in freshwater but also enhanced their combined toxicity [129]. Moreover, the surfaces of M/NPs provide a suitable solid phase to concentrate metal ions in aqueous solutions for the precipitation or crystallization of metal salts and oxyhydroxides [130]. In real scenarios, two or three of the above mechanisms might be involved in the interactions of M/NPs with metal ions.

Metal ions in the eco–corona of M/NPs may alter the physicochemical properties of M/NPs and further impact their adverse outcome pathways (AOPs), for example, cellular uptake [131, 132], distribution [131], interaction targets [132], and metabolism [133]. A report by Ramirezet al*.* [134] indicated that cation concentrations affect the surface properties and aggregation dynamics of PS NPs. Consistently, Abe et al*.* [135] discovered that divalent cations, in particular, Ca^2+^ and Mg^2+^ ions, can neutralize M/NP electrostatic stabilization that is enhanced by NOMs and induce aggregation through cation bridging and charge neutralization mechanisms.

In aquatic environments, M/NPs can be initially ingested by various aquatic organisms such as zooplankton [136], mussels [137], bivalve mollusks [138] and phytoplankton algae [139], and can accumulate in high aquatic organisms (*e.g., *fish) through the food chain. Interestingly, the formation of coronas on M/NPs may antagonize the hazardous effects of heavy metals in algae [140]. For instance, after binding to polyacrylonitrile polymer (PAN), the toxicity of Cu^2+^ in *Chlorella pyrenoidosa* was significantly ameliorated [141]. Fu et al*.* [142] found that the combination of Cu^2+^ (0.5 mg/L) and aged PVC (10 mg/L) even promotes the growth of Chlorella vulgaris. Coexposure of Cd^2+^, Cu^2+^ and Ni^2+^ with M/NPs showed negative impacts in earthworms. However, the combined toxicity of M/NPs and metal ions is still debatable, probably due to the differences in the tested M/NPs (*e.g., *sizes, compositions), metal species and animal models in different studies. For instance, small PS (0.5 μm) binding with metal ions shows a greater growth inhibition effect on microalgae than bare PS [143]; Cd^2+^–bearing MPs (2–4 μm) are more toxic in the cladoceran *Moina monogolica* Dadaya [144]. In addition, both synergistic and antagonistic effects on mercury adsorbed by M/NPs were reported. Fernández et al*.* [145] found that HDPE facilitated the ingestion of Hg^2+^ in mussels, but negligible bioaccumulation was detected in clams exposed to Hg^2+^–contaminated PE. Combined exposure to PS MPs and Cu^2+^ led to oxidative damage and inflammation in fish tissues and embryos, but the combination of Cd^2+^ and MPs had few toxic effects [146]. In mammalian systems, while free ions mainly pass through plasma membranes via ion channels or passive distributions and react with cytoplastic proteins [147], metal ions in the corona are internalized into lysosomes by endocytosis and interact with lysosomal proteins [148]. The persistent feature of M/NPs restricted the elimination of metal ions from the organism [149], resulting in prolonged bioavailability.

#### Natural organic matters

There is increasing evidence showing that M/NPs can sorb NOMs, which are mixtures of slightly water–soluble organic components consisting of humic substances (*e.g., *HA, fulvic acids (FA) and humins) and nonhumic fractions, such as amino acids, carbohydrates, and proteins [135]. The NOMs are widely present in aquatic and soil environments, with concentrations ranging from a few to hundreds of mg/L [150]. The HAs are the most studied NOMs and often display strong binding affinity with M/NPs by π–π and hydrophobic interactions [151]. In addition, since HA consists of sufficient carboxyl, hydroxyl and amine groups, the polar surface groups (*e.g., *–OH, –COOH, –NH_2_, –HSO_3_) on M/NPs may interact with HA by electrostatic interactions [152]. The intense hydrophobic and electrostatic forces may result in the formation of a hard corona of HA on the M/NP surfaces. However, there is no clear evidence supporting this speculation because the differentiation of hard and soft coronas is challenging.

The formation of NOM coronas on M/NPs may affect particle transportation, retention, and toxicity in the environment [153, 154]. Particles coated with NOM were generally observed to have lower attachment efficiencies than bare particles. Chen et al*.* [155] showed that HA–coated nanoparticles display reduced retention in saturated sand columns. The stability of positively charged PS increased significantly as NOM concentration increased from 5 to 20 ppm, but a reversal trend was examined in negatively charged NPs [156]. This is not surprising, as humic substances are commonly negatively charged at environmentally relevant pH values, and the adsorption of NOM onto positively charged M/NPs will reverse the surface charge. Formation of NOM coronas may slow the degradation of M/NPs. Wu et al*.* [157] recently found that photoaging of PP MPs was significantly inhibited in lake water compared with ultrapure water due to the coating of HA and FA on microplastic surfaces. This is because the humic/fulvic acid layer could act as a scavenger of oxidative radicals and optical light filters. In addition, the NOM corona may facilitate the transportation of M/NPs in porous media [158], seawater [159] and sands [160] and stabilize M/NPs by reducing aggregation and sedimentation [161]. In addition to environmental migration, NOM coronas showed differential effects in safety assessments of M/NPs. Liu et al*.* [162] systematically investigated the toxicological effects of three PS NPs with different functional groups (bare, amino and carboxyl) in the absence and presence of FA and HA. The formation of NOM coronas on PS NPs increased oxidative stress and membrane disruption in *S. obliquus* cells. The hazard effects were dependent on the concentrations and types of both M/NPs and NOM. Fadare et al*.* [163] investigated the impacts of NOM coronas on the toxicity of FA–coated NPs in *Daphnia magna*. The presence of HA resulted in the mitigation of gene expression, whereas significantly higher upregulation of all the genes was observed in *Daphnia magna* exposed to FA–coated NPs. Short–term exposure to HA–coated PS NPs elicited an immunomodulatory response, with activation of steroidogenic stress–related pathways in European seabass [164], but no notable alteration in inflammatory markers was found, indicating a protective anti–inflammatory effect of HA. Consistently, Wu et al*.* [165] showed an amelioration effect of HA in a toxicity test of PS NPs. Four PS NPs with different functional groups and charges were collected to investigate the effect of HA on particle aggregation behavior and toxicity. The results showed that HA exerted a stabilizing effect on three negatively charged NPs. The presence of HA effectively reduced the toxicity of PS and positively charged p–PS–NH_2_, as the survival rates of *Daphnia magna* increased from 15 to 45–95% and 100%, respectively [165].

#### Persistent organic pollutants

Persistent organic pollutants (POPs) are a class of organic compounds that are resistant to environmental degradation and have detrimental effects on the environment and human health [166]. Given that POPs and M/NPs are all from the wastes of engineered products, they may coincidentally exist in some specific areas, such as farmland, fishpond, and raw sewage [167]. M/NPs may stably adsorb hydrophobic POPs such as polycyclic aromatic hydrocarbons (PAHs) [168], polychlorinated biphenyls (PCBs) [169] and dichlorodiphenyltrichloroethane (DDT) [170] on their surfaces to form surface coronas. Hydrophobic interactions, halogen bonding, electrostatic interactions, and hydrogen bonding may be responsible for the binding of POPs to M/NPs [171]. For this reason, it is important not only to assess the occurrence and fate of M/NP binding with POPs in the environment but also to study the role of M/NPs as carriers of POPs [172]. Calculation results suggested that the delivery effect of M/NPs toward organic chemicals is limited in biological systems. Gouin et al*.* [173] used a thermodynamic approach to decipher the relationships between the physicochemical properties of organic chemicals and MPs. It was found that chemicals with log K(OW) > 5 could partition > 1% to PE. Foodweb model results suggested that the body burden concentrations may decline for nonpolar organic chemicals with log K(OW) between 5.5 and 6.5 after binding with MPs. Consistently, the bioavailability of phenanthrene (Phe) and 17α–ethinylestradiol (EE2) was reduced by 33% and 48%, respectively, after association with MPs [174]. Cormier et al*.* [175] investigated the adsorption processes of perfluorooctanesulfonate (PFOS) on four PE MPs with different size ranges. The adsorption capacity of PFOS increased with decreasing particle size and continuous binding of PFOS on particle surfaces over six months. Ma et al*.* [176] selected triclosan (TCS) as a model pollutant to investigate its interactions with small and large PVC MPs. Small PVC particles showed higher distribution coefficient values of TCS (1.35 L/g *v.s.* 1.05 L/g) and stronger adsorption capacity (12.7 mg/g *v.s.* 8.98 mg/g) than large PVC MPs. This is not surprising, as small M/NPs often have a higher specific surface area, better suspension, and stronger hydrophobicity. The initial pH value and salinity of the solution are critical factors dictating the adsorption process. Yeo et al*.* [177] found that the sorption of TCS on PS is higher within the pH range of 3.0–6.0 but is not affected by temperature at 288–318 K. Sørensen et al*.* [178] studied the sorption kinetics of two model PAHs (fluoranthene and phenanthrene) to PE and PS MP surfaces in natural seawater at 10 and 20 ℃. Temperature–dependent sorption was profiled. In terms of the sorption mechanism, PAH sorption behavior could be fitted by a monolayer sorption model at low PAH concentrations, whereas multilayer sorption was detected at higher concentrations [179].

These calculation or simulation results were confirmed in M/NPs collected from real environments. Zhang et al*.* [180] analyzed the MPs in sediment samples from deep–sea locations (4601–5732 m) of the western Pacific Ocean by micro-Fourier transform infrared spectroscopy (FTIR) and found a strong correlation (*p* = 0.016) between the concentrations of PCBs and MP distributions in sediments. This finding confirms that POPs exist in the surface corona of M/NPs even in deep–sea ecosystems. Both PCBs and PBDEs were observed in buoyant MPs collected in surface water at 27 locations in the Pacific Ocean around the coast of Japan, and their concentrations were 0.04–124 ng/g and 0–2158 ng/g, respectively [177]. The PCBs and organochlorine pesticides (OCPs) were detected in microplastic samples collected in six “clean” Italian minor islands and two “polluted” areas near the mouths of two major Italian rivers [181]. These findings indicated that POPs broadly coexist with M/NPs in the environment and become an important constituent of M/NP coronas.

Most toxicity studies showed that M/NP adsorption significantly reduced the bioavailability of POPs and adverse effects. Besseling et al*.* [182] provided the first study of plastic effects on benthic organisms, including the transfer of 19 PCBs in Arenicola marina. PAHs and M/NPs were co-exposed to represent the environmentally relevant exposure scenarios. M/NP sorption resulted in a corresponding reduction in free PAHs and amelioration of lethality and bioaccumulation, indicating that only freely dissolved PAHs are available to copepods under co-exposure conditions [178]. Gerdes et al*.* [183] evaluated the effect of microplastics on PCB removal in the cladoceran *Daphnia magna* exposed to a high body burden of PCBs 18, 40, 128 and 209. In the Daphnias fed microplastics, PCB 209 was removed efficiently, while no differences were detected for other PCBs. Grigorakis et al*.* [184] studied the diet assimilation efficiencies (AEs) of PCBs absorbed to MPs in goldfish (Carassius auratus). Compared to PCBs directly mixed in food, microplastic–bound PCBs had fewer AEs (13.36% *v.s.* 51.64%). Batel et al*.* [185] studied the fate, transfer and accumulation of microplastic–associated POPs, including PAHs and benzotalpyrene (BaP), in gills and embryos by fluorophore labeling. The biotransfer of BaP was visualized by fluorescence microscopy. A significant BaP signal was detected in gill filaments and arches as well as zebrafish embryos after incubation. While MPs did not release sufficient BaP to induce morphological changes in fish embryos, free waterborne BaP did induce effects in embryos. Heinrich et al*.* [186] investigated this in RTL–W1 cells exposed to 7–ethoxyresorufin–O–deethylase (EROD) adsorbed on three PE MPs. As a result, the bioavailability of EROD was reduced up to 79%; the activity was also significantly reduced in the presence of MPs. Scopetani *et* al.’s study showed that M/NPs could act both as carriers and scavengers for the bioaccumulation of organic pollutants (*i.e.,*PBDEs), suggesting that chemical leaching from MPs has a limited impact on biota [124]. However, this antagonizing effect may depend on the properties and doses of M/NPs. Jeong et al*.* observed enhanced toxicity of 2,2',4,4'–tetrabromodiphenyl ether and triclosan in B. koreanus during coexposure to nanosized plastics, probably because NPs had higher accumulation and more oxidative stress–induced damage than MPs [187].

### Bio-coronas

The M/NPs often initially interact with natural substances in environments to form eco–corona. However, once these particulates encounter biological systems, they may interact with abundant biomolecules, including proteins, lipids, and nucleic acids to replace some of the constituents in eco–coronas and form bio-coronas on surfaces [24]. These biomolecules are either primarily extracellular polymeric substances (EPS) from organism metabolism or intracellular components. The driving forces of bio-corona formation are also hydrophobic and electrostatic interactions [163]. In most cases, environmental matter and biomolecules may coexist in the surface corona to confer a new biological identity for M/NPs. During the past decade, bio-corona formation has attracted substantial research interest in the field of nanomedicine because the corona structure may significantly affect the pharmacological/toxicological profiles of some engineered nanosized drug carriers [188], imaging agents [189] and/or transplants [190]. The bioavailability, immune recognition, cellular internalization, cellular distributions, and cytotoxicity are largely dependent on the constituents of bio-coronas [191]. To comprehensively assess the biosafety of M/NPs for health protection, the formation of bio-corona as a critical determinant needs more exploration. Three types of biomolecules, including proteins, lipids and nucleic acids, have been identified in surface coronas (Table 1).

**Table 1:** The identified bio-coronas on M/NPs

Bio-corona composition	Source	M/NPs	References
Proteins	Physiological fluid of Daphnia magna	PS–NH_2_	[163]
Proteins	Cell culture medium for 24 h	PS	[190]
Mucin	Mucous layer in lung epithelial	PS@Bap NPs	[194]
MgC1q6 protein	Serum soluble components	PS–NH_2_	[191]
Carbohydrates and Proteins	EPS from marine diatom Phaeodactylum tricornutum	PS–COOH	[192]
Lipids and Proteins	Alveolar fluid	PS PET PP PE PVC	[195]
Biofilm consist of nitrogen– and sulfur–related substances	Staphylococcus aureus	Nontreated and amino acid–treated plastic traces	[196]
Nucleic acids	Coral microbiome	PE PP PS PVC	[197]
Toxins and ARGs	Bacteria	PE PP	[198–200]

#### Proteins

Each physiological compartment in biological systems has its own distinct set of proteins that interact in a unique way with M/NPs. The formula of NOM, metal ions or POPs in the eco–corona of M/NPs may significantly impact the bio-corona constitution. For instance, the adsorption of proteins on NPs was increased in the presence of FA in *D. magna* culture medium, while the presence of HA led to a decline in protein adsorption [163]. Grassi et al*.* [192] employed EPS from the marine diatom *Phaeodactylum tricornutum* to study the changes in eco–corona on PS–COOH nanoplastic surfaces. EPS significantly reduced the PS–COOH aggregation rate and caused the formation of a complex bio-corona consisting of carbohydrates and proteins. Sodium dodecyl sulfate–polyacrylamide gel electrophoresis (SDS–PAGE) analysis of proteins adsorbed on PS–COOH showed abundant proteins in the molecular weight range of 30–100 kDa.

**Fig. 4:**
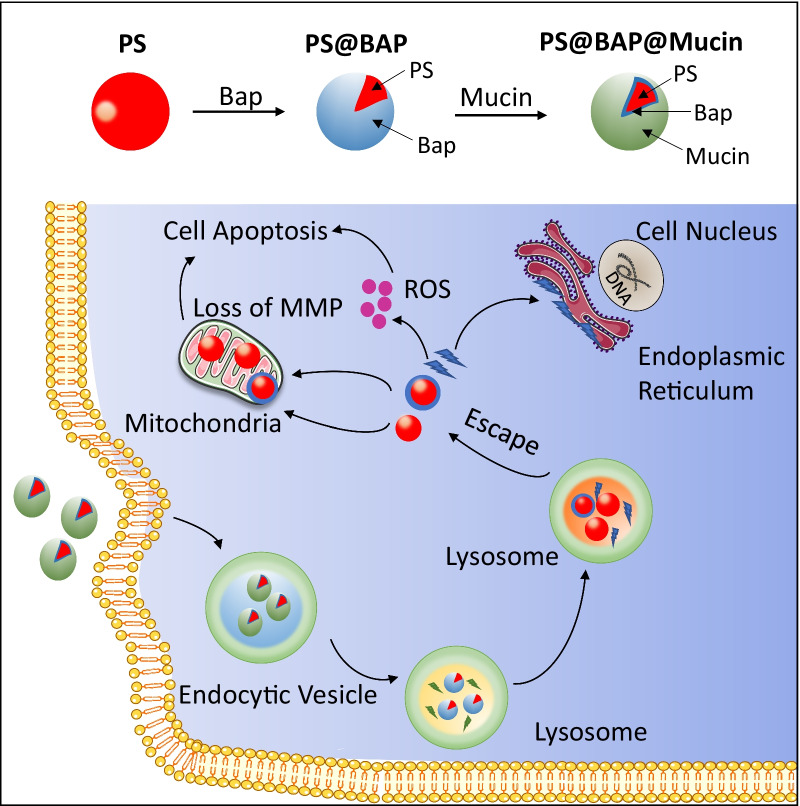
The formation of a protein corona (mucin) on nanoplastics alters the intracellular fate and final destinations of the particles. PS@Bap with mucin corona was internalized into lysosomes in A549 cells via endocytosis. The mucin corona is stable at the early stages and degrades in the maturation of lysosomes, altering the intracellular trafficking of PS@Bap and the release of Bap from PS. Adapted and reprinted with permission from [194]**.**

In mammals, ingestion is the major entry route for most M/NPs. However, NPs may be inhaled into the respiratory system since they may form stable aerosols in air [193]. Taking PS–benzopyrene (PS@Bap) nanoparticles as an example, Ji et al. found that the PS@Bap particles could be inhaled by the respiratory system, interacted with the mucous layer in the lung epithelium and formed a mucin corona on the surfaces. Further study showed that although the mucin corona is stable at the early stages of PS@Bap endocytosis in A549 cells, it degrades during the maturation of endosomes into lysosomes [194]. In addition, the mucin corona enhances the cellular uptake of PS@Bap but delays the intracellular trafficking of PS@Bap as well as the release of Bap from PS, resulting in reduced ROS generation, mitochondrial impairment and apoptotic cell death (Fig. 4).

#### Lipids

Lipids are an essential component of biomembranes (*e.g., *vesicles), which are involved in a variety of physiological processes, including carbon storage, tissue protection, and resistance to exogenous substances [201]. Compared to proteins, the biological functions and structures of lipids are poorly understood. There are diverse lipid derivatives in biological systems, such as waxes, triacyl–glycerides, phospholipids, sphingolipids and glycolipids [202], which have differences in hydrophobic tails and hydrophilic heads. By interacting with M/NPs, lipids, especially unsaturated lipids, may be oxidized, with the subsequent generation of –OOH, –OH, –O–, and hypochlorite moieties on the hydrophobic tails of lipids. These functionalized lipids often take part in many biological processes. For instance, lipid peroxides are important hazard signals that activate ferroptosis, which was recently identified in iron–dependent programmed cell death [203, 204]. Lipid oxides have different biological functions due to their distinct structures, sites and quantities of oxidation [205]. Therefore, the analysis and identification of the composition and structure information of functionalized lipids is of great significance for the in–depth analysis of their biological functions.

Digestion and absorption of lipids in the intestinal tract are the key processes for energy and fat biosynthesis, which play key roles in the early growth of organisms [206]. After ingestion, M/NPs were primarily distributed in the intestine and could induce intestinal dysfunction. A few studies revealed that M/NPs disturbed lipid metabolism in aquatic organisms by causing intestinal microbiota dysbiosis or affecting the relevant enzymatic activities [207]. Recently, an in vitro study found that PS MPs inhibited lipase activities in a simulated human gastrointestinal system, reducing lipid digestion and bioavailability of dietary lipids [208]. However, the impact of MPs on the whole lipid metabolism process (*e.g., *intestinal digestion, bile acid secretion, triglyceride resynthesis and transport) in vivo remains largely unknown. In terms of respiratory exposure, M/NPs may interact with a thin layer of lipids and lung surfactants in alveoli. Theodorou et al*.* [209] showed that the formation of phospholipid coronas on ZnO nanowires preincubated with Curosurf® (a natural porcine pulmonary surfactant) could significantly increase the cellular uptake of the nanowires within human alveolar epithelial type 1–like cells (TT1) while decreasing intracellular dissolution and thus impacting cell death. However, few attempts have been made to investigate the detailed interactions between M/NPs and lipids, and critical data on the types and quantities of lipids associated with M/NPs are lacking.

#### Nucleic acids

Nucleic acids, including DNA and RNA, are the primary information–carrying molecules in organisms. A recent study demonstrated the presence of fish pathogens and human–associated bacteria on marine plastics from Norway [198]. Genes encoding toxins, hemolysins and adhesion factors as well as new variants of antibiotic resistance genes (ARGs) were identified in a salmonicida isolate obtained from marine plastics. Their study strengthens the notion that plastic debris may serve as vectors for the transport of fish pathogens as well as other opportunistic human pathogens in the marine environment. In aquatic systems, M/NPs provide a suitable solid support for the growth of microorganisms to generate biofilms. For example, PE and PP polymers (335 mm) from the Jiaxing River, China, were shown to have selective enrichment of 25 ARGs, most of which were linked with sulfonamide resistance gene (SA), aminoglycoside resistance genes (AMG), tetracycline resistance genes (TC), macrolide–lincosamide–streptogramin resistance genes (ML), and abundance of florfenicol resistance genes [199]. On PE films (5 mm) incubated (28 d) in waters from Taihu Lake, China, 8 ARGs (sul1, sulA/folP, ermE, ermF, tetA, tetC, tetW, tetX) were identified. This study concluded that MPs in the river had more ARGs than those in the estuary and seawater. The adsorption of antibiotic resistance elements (AREs), including ARGs and mobile genetic elements (MGEs), on M/NP surfaces has induced substantial concerns about the spread of antimicrobial resistance [200].

## Analytical methods for coronas on M/NPs

**Fig. 5:**
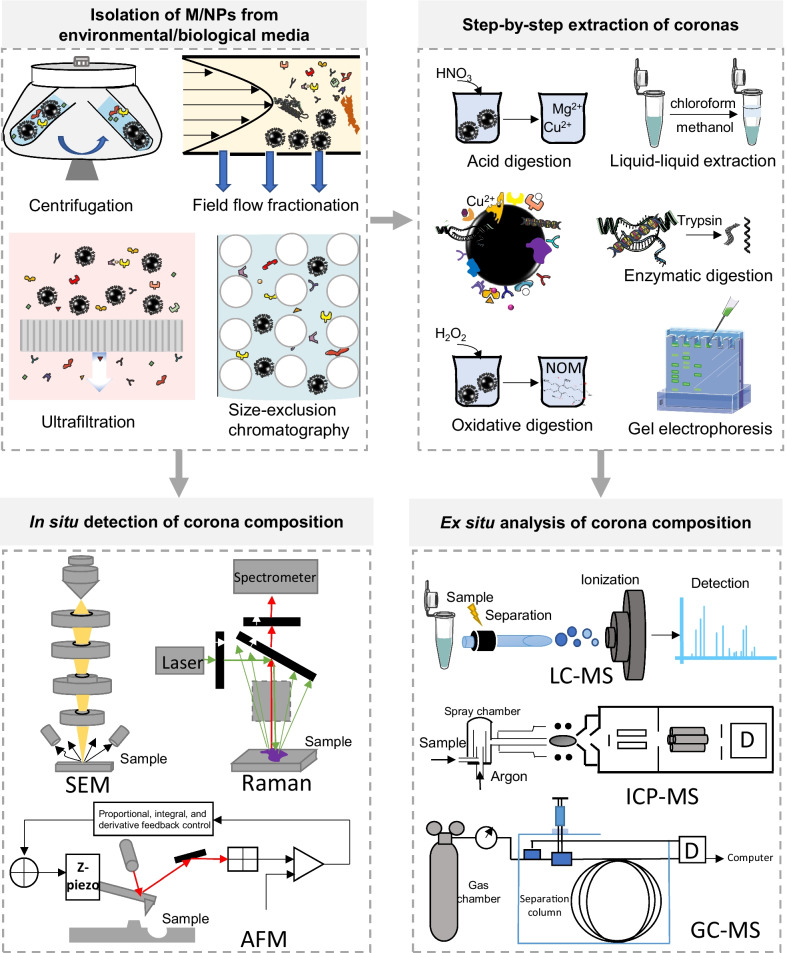
Systematic analysis strategy for a deep quantitative analysis of the corona structure on M/NPs. M/NPs can be isolated from environmental/biological media by centrifugation, ultrafiltration, field flow fractionation, etc. The coronas on the M/NPs can be detected both by in situ and ex situ methods. For in situ analysis, the corona structures are directly interpreted by some microscopic and spectroscopic techniques, such as SEM, AFM, and Raman spectroscopy. For ex situ analysis, the coronas need to be separated from the M/NPs by layer–by–layer extraction methods and detected by various MS–based methods, including LC–MS, ICP–MS, and GC–MS.

To date, it remains largely unexplored how corona formation on M/NPs determines their biological effects in organisms. Gaining insight into the biological impacts of M/NPs requires identification and quantification of the corona constitutions, raising challenges for analytical methods. In situ analysis was initially proposed to interpret the corona structures by material characterization techniques, such as ultraviolet-visible spectroscopy (UV-vis) [210], Raman spectroscopy [211], FTIR [212], X-ray photoelectron spectroscopy (XPS) [213], Auger electron spectroscopy (AES) [214], atomic force microscopy (AFM) [215], time of flight secondary ion mass spectrometry (TOF-SIMS) [216], or energy–dispersive X-ray (EDX) [217] analysis. However, most of these methods are not suitable to identify all molecular constituents in coronas. For complex M/NP samples, an *ex–situ* systematic analysis strategy is required for a deep quantitative analysis of the corona structures (Fig. 5), including isolation of M/NPs from environmental/biological media, layer–by–layer extraction of coronas, identification and quantification of the constituents.

### Isolation of M/NPs from environmental/biological media

The isolation of M/NPs usually depends on the sample matrix. For M/NPs in clean aquatic or gas environments, this step can simply be achieved by filtration or centrifugation [218]. Nevertheless, more sophisticated techniques, such as ultracentrifugation [219], ultrafiltration [220], cloud point extraction (CPE) [221], and continuous flow centrifugation (CFC) [222], may be needed for the separation of M/NPs from complex matrices. Ultracentrifugation is a widening of benchtop centrifugation, which spins samples at exceptionally high speeds and separates small particles in a solution by their size, shape and density. Since the first use of ultracentrifugation in the separation of gold nanoparticles, it has been one of the most popular methods in nanoscience [223]. Recently, several studies reported the application of ultracentrifugation in the separation of plastic particles from aqueous matrix [224] or sediments [225, 226]. Before separation, matrix digestion with acid or alkali is usually considered a useful step to remove unbonded organic matter and improve the separation efficiency in ultracentrifugation; however, it may lead to the loss of organic eco–corona and bio-corona [227, 228]. Enzymatic digestion is also a commonly used method to remove macrobiomolecules, such as protein and DNA, in organic matrices. Enzymatic digestion can be performed for the measurement of small molecules in eco– and bio-coronas but will cause the loss of proteins [229]. Attempts have been made to facilitate the isolation of M/NPs by additives to tune solution density and control centrifugation force and times. Löder et al. [230] and Thompson et al*.* [8] demonstrated that the introduction of an appropriate salt (> 1.4 g/cm^3^) in MP aqueous samples could be used to retrieve M/NPs because most M/NPs have a density ranging from 0.8 to 1.4 g/cm^3^. Cai et al*.*’s [224] study indicated that the theoretical centrifugal time at a speed of 20,000 rpm for 2 h could completely assure sediment of 600–1000 nm spherical PS from environmental water samples. However, the presence of other particulates in M/NP suspensions may significantly affect the purity of isolated M/NPs by ultracentrifugation. Therefore, more separation methods, *e.g., *magnetic separation [231], field flow fractionation [232], and size–exclusion chromatography (SEC) [233], have been developed for the efficient isolation of M/NPs. For the efficient collection of M/NPs in real environmental/biological samples, combined separation methods are recommended.

### Extraction of coronas from M/NPs

The composition of coronas on M/NPs can be extremely complex due to the high diversity of the matrix in the environment. The coronas are usually complexes of metal ions, inorganic salts, proteins, DNAs, lipids, HA and other small organic compounds. Obviously, it is impossible to measure both the amount and composition of all substances in the corona structure by a single determination. A more feasible way is to extract each constituent of coronas step by step and analyze the compounds by different methods. Separation of the coronas from M/NPs can be achieved by a rational design of sample pretreatment methods according to the chemical properties of the target compounds. For instance, the absorbed small organic substance could be decoupled from M/NPs by solvent extraction, such as H_2_O_2_, Fenton’s reagent, and alkaline digestion with NaOH and KOH [234], while inorganic salts and metal cations could be directly eluted in aqueous solvent after digestion of the particles by 68% HNO_3_ (Aristar grade) or HCl–HNO_3_–HF–HClO_4_ acids [235, 236]. Many of the approaches for elution of small molecules from biofluids or tissues could be applied in the extraction of corona constituents. One of the successive ways is to wash the particles by solvents ranging from polar (such as water) to less polar solvents (such as methanol, hexane and chloroform) [237]. The choice of solvents mainly depends on the type of molecules being extracted. Water or ethanol are more suitable for polar molecules, whereas hexane or chloroform are more suitable for nonpolar compounds. Biphasic extraction, such as the Bligh–Dyer, Folch and Matyash methods, is suitable in cases where both polar and nonpolar coronas need to be collected [238]. The pH values of the extraction solvent could be adjusted to alter the charge status of coronas or promote the hydrolysis of bonds between M/NPs and coronas [239]. Despite these general rules for corona extraction, explorations are still desired to optimize the extraction method for analysis of the whole corona rather than a small subset of compounds bound with M/NPs.

In addition to small molecules, large biomolecules, including proteins, polysaccharides and nucleic acids, are another major component of coronas. Of them, the protein corona is the most studied and can be extracted by buffer containing sodium dodecyl sulfate and other ionic detergents that have the ability to solubilize proteins [240]. The total protein content in the extraction buffers can be quantified by Bradford [241], BCA assays [242] or UV–vis [243]. Other separation methods, such as western blotting [244], gel electrophoresis [245], and SEC [246], can also be applied to isolate the proteins for further detection. Recently, Grassi et al*.* studied coronas on carboxylated PS NPs, which were incubated with EPS. A separation of the coronas by SEC revealed four main distinct groups of peaks, containing high (> 100 kDa) to low molecular weight (20 kDa) species with high chemical heterogeneity [192]. Another classical approach for characterization of the protein coronas on NPs is enzymatic digestion of the attached proteins and then quantification of the peptides in digests by mass spectrometry, which is subsequently submitted to a protein database for protein identification. To date, high–resolution mass spectrometry has been the most recommended state–of–the–art method for the identification of protein coronas. There are a series of review articles that can be referenced to obtain deeper knowledge on this topic [22, 247–249].

### Identification and quantification of the coronas

Since the first report on nanocoronas, many analytical techniques have been implemented in the characterization of the coronas composition on various engineered nanomaterials. In recent years, some of these techniques, including microscopic, spectroscopic, and mass spectrometry–based methods, have been explored for identifying the coronas on plastic debris. Table 2 lists the utilities of these techniques to determine the corona information of M/NPs, their strengths, and limitations. Obviously, no single technique is sufficient to address all the questions about coronas on M/NPs on its own. In most cases, combined detection methods are recommended. The methods for corona analysis can be mainly divided into two categories.

**Table 2:** Potential analytical techniques for the characterization of corona formation on M/NPs, their strengths and limitations

Techniques	Corona Information	Sample states	Strengths	Limitations
UV–vis	Binding of protein corona to M/NPs	Particles	High reproducible and precision. nondestructive	Not suitable for qualification, susceptible to sample matrix, low sensitivity
DLS	Binding of coronas to M/NPs	Particles	Rapid and reproducible measurement of many nanoparticles, nondestructive	Not suitable for qualification and quantification, not suitable for heterogeneous particles
Fluorescence microscopy	Binding of coronas to M/NPs	Particles	High sensitive and good for real–time detection	Requirements for fluorescence labeling
TEM	Interactions between M/NPs and corona	Particles	High spatial resolution	Not suitable for qualification and quantification
SEM	Interactions between M/NPs and corona	Particles	High spatial resolution	Low particle population, poor qualification and quantification
AFM	Interactions between M/NPs and corona	Particles	High spatial resolution, possibility for 3D imaging	Poor efficiency
EDX	Elemental, especially metal elemental distribution	Particles	High efficiency	Not suitable for organic elements
Raman	Structures of organic substances, such as natural organic matters, lipids, persistent organic pollutants	Particles	Broad coverage of both organic and inorganic species	Low spatial resolution
FTIR	Structures of organic substances, such as natural organic matters, lipids, persistent organic pollutants	Particles	Good repeatability, high flexibility	Low accuracy and sensitivity, not available for inorganic species
ICP–MS	Compositions of metal or metal ions	Acid digested solution	High sensitivity and accuracy, quantification	Not suitable for organic compounds
TOF–SIMS	Structures of organic substances, such as natural organic matters, lipids, persistent organic pollutants, proteins	Particles	3D profile with high mass resolution and spatial resolution	Poor quantification, not suitable for unknown compounds, requirements for samples
GC–MS	Compositions of hydrophobic and volatile organic compounds, such as flame retardants, lubricants and plasticizers and persistent organic pollutants	Solvent extracted solution	High sensitivity and reproducibility	Not suitable for thermally labile compounds
LC–MS	Compositions of thermally labile coronas, such as proteins, lipids, peptides	Enzyme digested or solvent extracted solution	Broad coverage of compounds, high sensitivity and accuracy	Requirements for multistep sample pretreatment, ex situ analysis
CE–MS	Compositions of thermally labile coronas, such as proteins and metabolites	Enzyme digested or solvent extracted solution	High resolution and selectivity	Not suitable for direct analysis of high molecular proteins, limited loading capacity

#### Microscopic and spectroscopic methods

Microscopic and spectroscopic methods are integral parts of techniques for nanoparticle characterization. Some of these methods, such as UV–vis, DLS, TEM, SEM, EDX, AFM, Raman or FTIR, are also commonly used for in situ deciphering corona formation on particle surfaces. For example, UV–vis can be used to evaluate the binding of proteins to particles by assessing the absorption peak changes before and after binding [250, 251]. Corona formation on M/NPs could also be determined by measuring the changes in hydrodynamic size of M/NPs using DLS [252], fluorescence microscopy [253]. However, such measurements are not suitable when particles are colloidally unstable and exhibit a wide size distribution. TEM, SEM and AFM can provide high–resolution images of M/NPs for visualizing the interactions between plastics and corona by morphology changes [254, 255]. By coupling with EDS, TEM and SEM can reveal the elemental distribution on plastic particle surfaces, which is a convincing technique to analyze metal elements in the corona [255]. Although phosphorus, oxygen and carbon elements are also detectable, this technique has limitations in differentiating adsorbed organic substances from plastic surfaces. Instead, FTIR and Raman spectroscopy are more suitable to detect the organic substances in corona structures. For instance, FA, lipids and sodium dodecyl sulfate were successfully detected on nano–PS by surface–enhanced Raman scattering in the study of Zhang et al*.* [256]. However, both FTIR and Raman spectroscopy are incapable of directly visualizing M/NPs. Recent advances in characterization techniques, *e.g., *AFM–IR, combine the advantages of microscopic and spectroscopic methods and may offer opportunities for the identification of organic substances in the corona structure of a single NP [257]. Among other optical microscopy techniques, hyperspectral microscopy in dark-field [258] has recently been introduced as an effective method to detect and identify nanoplastics down to 100 nm [259, 260]. Supplemented by artificial intelligence [261] and machine learning [262–264], this technique may hold promise in spectral characterization of adlayers deposited onto microplastics. Future work is needed to evaluate the feasibility of this technology in characterization of coronas on microplastics. However, all these microscopic and spectroscopic techniques are not suitable for a comprehensive interpretation of each constituent in corona structures. To acquire the fingerprints of M/NP coronas, mass spectrometry–based methods should be considered.

#### Mass spectrometry–based methods

MS has been predominantly used as a sensitive and high–resolution technique to examine the chemical composition of coronas. It can be used to detect molecular weights or structures via the mass–to–charge ratio of fragments of metal ions, small chemicals and biomolecules, covering almost all known constituents in corona structures. Currently, various MS–based procedures, including inductively coupled plasma–mass spectrometry (ICP–MS), TOF–SIMS, gas chromatography–mass spectrometry (GC–MS), capillary electrophoresis–mass spectrometry (CE–MS) and liquid chromatography–mass spectrometry (LC–MS), have been explored for the examination of coronas on M/NPs. ICP–MS has been extensively used for the measurement of inorganic elements, particularly trace metal contaminants, in M/NP coronas. For instance, Qiao et al*.* [265] used ICP–MS to detect Cu uptake in zebrafish and found that small–sized MPs deliver more Cu. This technique has also been successfully exploited to examine the transport of adsorbed heavy metals (Pb, Cd, and Zn) by PET MPs in wheat [132]. Although trace levels (~ 0.3 ppb) of metal elements could be well quantified by ICP–MS analysis, they could merely reflect the average metal levels of the bulk particle population. While each M/NP in nature has its own individual physicochemical properties and may form distinct coronas, the interpretation of coronas on a single plastic particle is meaningful. In 2003, Degueldre developed single particle (sp)ICP–MS, which measures the mass of recorded elements in individual particles and the total particle number concentration [266]. Recently, Bolea–Fernandez et al*.* [267] successfully detected ^13^C–labeled and lanthanide–doped PS beads by single particle–ICP–MS. However, neither ICP–MS nor SP–ICP–MS can characterize the molecular structure of organic coronas. Compared to ICP–MS, TOF–SIMS is more suitable for the characterization of organic compounds. TOF–SIMS can determine the element and molecular fragments by the SIMS peak, which is produced by measuring the exact mass and intensity of secondary ions and ion clusters emitted from a solid surface with a finely focused ion beam. Basically, TOF–SIMS is a surface analysis technique that functions in a manner analogous to SEM/EDS. However, the depth of analysis for TOF–SIMS is less than 2 nm, which is better suited for the analysis of ultrathin layers and nanoscale samples when compared to SEM/EDS. In addition, TOF–SIMS can provide a unique 3D profile with high mass resolution and high spatial resolution and is an ideal technique for directly imaging organic constituents in coronas. Neunzehn et al*.* [268] exploited this technique to detect different nanoscale protein coatings on gold nanoparticles, but few attempts have been made on M/NPs. Notably, TOF–SIMS is not suitable for unknown compounds. In addition, this technique is sensitive to sample preparation and time–consuming in data interpretation and has poor performance in quantification analysis.

MS can also be coupled with various separation techniques, such as GC, LC and CE, to acquire massive information of a complex corona. GC–MS is one of the most useful techniques for monitoring highly hydrophobic and volatile organic compounds and has been extensively used for monitoring plastic additives (flame retardants, lubricants and plasticizers) and microplastic–adsorbed organic contaminants, such as OCPs, PAHs, PCBs, and organophosphorous pesticides (OPPs) [269]. León et al*.* [270] detected 91 organic pollutants, including 17 PAHs, 7 PCBs, 54 pesticides, and 5 plastic additives, by GC–MS in plastic debris from the Mar Menor coastal lagoon in southeastern Spain. The GC–MS allows the analysis and detection of tiny amounts of organic substances with high sensitivity and reproducibility, whereas it is not suitable for the analysis of thermally labile compounds. Alternatively, LC–MS is more commonly used to investigate the thermally labile constituents in coronas, including proteins, lipids, peptides. LC–MS can provide qualitative and quantitative information regarding the complex mixture of coronas and has been considered the first choice for protein corona studies. Fadare et al*.* [163] exploited this technique to analyze the coronas on M/NPs after interacting with humic substances and identified a total of 1004 proteins, with 281 and 723 unique proteins for eco–corona and bio-corona, respectively. In addition, CE–MS has recently been introduced as a new technique for the characterization of protein and metabolite coronas on nanomaterials [271]. Nevertheless, full interpretation of coronas on M/NPs is still a major challenge, which may require a rational combination of several analytical techniques.

## Summary and Perspectives

Spreading and accumulation of M/NPs have led to increasing interaction opportunities of M/NPs with substances in environmental and biological media. Given the hydrophobic, inert and persistent features of M/NPs, corona formation is a prominent process that is highly related to the migration, uptake, distribution, metabolism, clearance and toxicity of M/NPs. Although substantial evidence has demonstrated the coexistence of environmental/biological species and M/NPs, most studies merely assess the impacts of one or two substances on M/NP toxicity rather than the whole integrates. While concerns have been raised about the environmental and health risks of M/NPs and substantial efforts have been made in this research area, we recommend serious consideration of the role of surface coronas in safety assessments. Different from conventional toxins, packages on M/NPs rather than bare particles are more dangerous. The adsorption process on M/NPs may impact the bioavailability, entry routes, distribution and clearance of corona constituents in biological systems. Given that environmental and biological transport may significantly alter the physicochemical properties of M/NPs, each M/NP particle may have a distinct corona formula that generates a unique identity. Full interpretation of a corona structure is an extreme challenge and may require a rational combination of different analytical methods. All considered, corona formation in M/NP safety assessment is a new research field that faces challenges and opportunities.

- Rational combinations of analytical methods or exploration of new analytical technologies are extremely desired for a comprehensive interpretation of corona compositions.
- In addition to chemical composition, the advanced space structures of coronas, especially for bio-coronas, have rarely been explored.
- The transportation of M/NPs in a biological system may lead to a dynamic evolution of corona structures, which may further elicit molecular initiating events and adverse outcomes. The connections among these events are largely unknown.
- In situ detection of coronas on M/NPs in biological systems is a major challenge. Attempts should be made to use multiple independent labeling strategies (*e.g., *fluorescent and radiological labeling) for differentiation of the corona institutes and M/NPs in cells or animals.
- Big data analysis or machine learning is recommended to decipher the connections between corona structures and toxic effects.

Overcoming these challenges may require the exploration of interdisciplinary approaches in toxicology, materials science and analytical chemistry. A full understanding of the impacts of the corona on toxicity may facilitate the establishment of a predictive toxicology paradigm for risk assessments of M/NPs.

## Data Availability

Not applicable.
